# Pyruvate Accumulation Is the First Line of Cell Defense against Heat Stress in a Fungus

**DOI:** 10.1128/mBio.01284-17

**Published:** 2017-09-05

**Authors:** Xing Zhang, Raymond J. St. Leger, Weiguo Fang

**Affiliations:** a Institute of Microbiology, Zhejiang University, Hangzhou, China; b Institute of Insect Sciences, Zhejiang University, Hangzhou, China; c Department of Entomology, University of Maryland, College Park, Maryland, USA; University of Texas Health Science Center

**Keywords:** *Metarhizium robertsii*, entomopathogenic fungi, heat stress

## Abstract

Heat tolerance is well known to be key to fungal survival in many habitats, but our mechanistic understanding of how organisms adapt to heat stress is still incomplete. Using *Metarhizium robertsii*, an emerging model organism for assessing evolutionary processes, we report that pyruvate is in the vanguard of molecules that scavenge heat-induced reactive oxygen species (ROS). We show that, as well as inducing a rapid burst of ROS production, heat stress also downregulates genes for pyruvate consumption. The accumulating pyruvate is the fastest acting of several *M. robertsii* ROS scavengers, efficiently reducing protein carbonylation, stabilizing mitochondrial membrane potential, and promoting fungal growth. The acetate produced from pyruvate-ROS reactions itself causes acid stress, tolerance to which is regulated by Hog1 mitogen-activated protein kinase. Heat stress also induces pyruvate accumulation in several other fungi, suggesting that scavenging of heat-induced ROS by pyruvate is widespread.

## IMPORTANCE

Heat is a dangerous challenge for most organisms, as it denatures proteins and induces the production of ROS that inactivate proteins, lipid membranes, and DNA. How organisms respond to this stress is not fully understood. Using the experimentally tractable insect pathogen *Metarhizium robertsii* as a model organism, we show for the first time that heat stress induces pyruvate production and that this functions as the first line of defense against heat-induced ROS. Heat stress also induces rapid pyruvate accumulation in other fungi, suggesting that pyruvate is a common but unappreciated defense against stress.

## INTRODUCTION

Heat represents a challenging problem for the survival for most organisms (1, 2), including fungi, where population richness is largely determined by climate factors such as the mean annual temperature (3). Heat denatures proteins and induces the production of reactive oxygen species (ROS) that can chemically alter or inactivate proteins, lipid membranes, and DNA. Known defenses include trehalose and heat shock proteins (HSPs) that act complementarily at high temperatures by preventing protein aggregation, unfolding aggregated proteins, or targeting denatured proteins for degradation (4). The ROS-scavenging catalases and superoxide dismutases (SODs) and the protein repair enzymes TRX-TRR (thioredoxins-thioredoxin reductases) and GRX-GLR (glutaredoxins-glutathione reductases) also respond to heat stress (5). However, despite hundreds of relevant publications, our understanding of the fungal heat stress response is still incomplete (6).

The endophytic insect-pathogenic fungus *Metarhizium robertsii* (formerly *Metarhizium anisopliae*) is an emerging model for the assessment of fungal evolution in natural communities. It has a worldwide distribution from the arctic to the tropics (7, 8) and must therefore have the necessary molecular machinery to adapt to heat and cold stresses. A cold shock protein that contributes to the freezing tolerance of *M. robertsii* was the first of its kind characterized in fungi (9). However, a multiyear field trial of an *M. robertsii* strain revealed that its ability to maintain large populations in temperate climates is determined by its tolerance to heat stress (8). The mechanisms of this tolerance are unknown. Here, we report that heat stress downregulates key pyruvate consumption genes in pyruvate metabolism pathways, allowing rapid accumulation of pyruvate. Using nonenzymatic reactions, the pyruvate scavenges ROS faster than other ROS scavengers (catalase, SOD, peroxiredoxin, and glutathione peroxidase). Pyruvate is thus in the vanguard of molecules that scavenge heat-induced ROS in *M. robertsii*. Pyruvate produced in response to hypoxia also functions as a scavenger of ROS in mammalian cells (10). Addition of pyruvate to cell suspensions of two microaerophilic protozoa (*Giardia intestinalis* and *Hexamita inflata*) facilitates their tolerance to oxidative stress by eliminating ROS (11). To our knowledge, this is the first study to document in any organism that heat treatment induces pyruvate accumulation. However, as heat also induces pyruvate accumulation in five other fungi, pyruvate scavenging of heat-induced ROS could be widespread.

## RESULTS

### Heat stress induces pyruvate accumulation.

**FIG 1:**
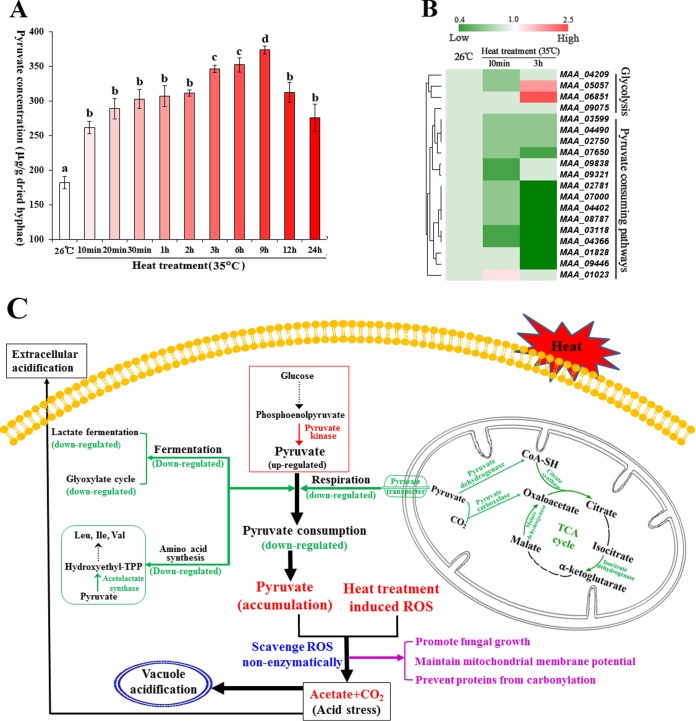
Heat treatment causes pyruvate accumulation. (A) Pyruvate concentrations in heat-treated hyphae. Values with different letters are significantly different (one-way ANOVA, *P* < 0.05). (B) The expression of pyruvate metabolism genes was changed by heat treatment. (C) A model showing how changes in the expression of pyruvate metabolism genes result in the accumulation of pyruvate, which nonenzymatically scavenges heat-induced ROS. Green, downregulated genes for pyruvate consumption; red, the single upregulated gene for pyruvate production, which was detected after 3 h of heat treatment. Mechanisms for tolerance to heat-induced acid stress are also shown. TPP, thiamine pyrophosphate.

The optimal temperature for *M. robertsii* is 26°C (7), and we found that its growth is significantly inhibited at 35°C and stopped at 37°C (see Fig. S1 in the supplemental material). To investigate how *M. robertsii* responds to heat stress, we compared the transcriptomes of hyphae grown for 3 h at 26 or 35°C in half-strength Sabouraud dextrose broth plus 1% yeast extract (1/2SDY). Transcriptome sequencing (RNA-Seq) revealed 2,124 differentially expressed genes (DEGs), from which 11 KEGG (Kyoto Encyclopedia of Genes and Genomes) pathways were enriched (Fig. S2A and B). Most noticeably, heat treatment caused downregulation of genes for pyruvate (an α-keto acid) consumption in the pyruvate metabolism pathway (Fig. S2C). We followed this up by quantifying pyruvate levels in hyphae and found that they increased ~1.5-fold (*F* = 12.08, *P* = 0.02) within 10 min following a transfer from 26 to 35°C and plateaued for ~2 h before increasing again to a peak at 9 h (Fig. 1A).

10.1128/mBio.01284-17.1 FIG S1  Growth of *M. robertsii* on PDA plates under heat stress. (A) Growth curve of *M. robertsii* on PDA plates at the optimal temperature (26°C) and under heat stress (35 or 37°C). The growth rates differed significantly (*t* test, *P* < 0.01) at ≥4 days postinoculation. (B) Colonies of *M. robertsii* on PDA plates at 26, 35, or 37°C. Pictures were taken at 14 days postinoculation. Bars, 10 mm. Presented are typical pictures representative of three repeats. Download FIG S1, JPG file, 0.4 MB. Copyright © 2017 Zhang et al. 2017 Zhang et al. This content is distributed under the terms of the Creative Commons Attribution 4.0 International license.

10.1128/mBio.01284-17.2 FIG S2  RNA-Seq analysis of the response of *M. robertsii* to heat stress. (A) Comparison of gene expression levels in hyphae grown at the optimal temperature (26°C) or heat treated (35°C) for 3 h. The expression level of each gene was normalized as RPKM. Genes up- and downregulated in heat-treated hyphae, compared to those in hyphae grown at 26°C, are red and green, respectively. Genes that were not differentially expressed in the two groups tested are blue. The *x* axis represents the log_10_ RPKM of control samples (26°C). The *y* axis represents the log_10_ RPKM of heat-treated samples. (B) Categories of 377 DEGs assigned in the KEGG database. (C) The pyruvate metabolism pathway generated by the KEGG enrichment analysis for the DEGs. Downregulated genes are green. Noted are the downregulated genes encoding enzymes involved in pyruvate consumption, i.e., the malate dehydrogenase (EC 1.1.1.40) in the TCA cycle, the subunit (EC 2.3.1.12) in the pyruvate dehydrogenase complex, and the pyruvate carboxylase (EC 6.4.1.1). Download FIG S2, JPG file, 1.1 MB. Copyright © 2017 Zhang et al. 2017 Zhang et al. This content is distributed under the terms of the Creative Commons Attribution 4.0 International license.

We used quantitative reverse transcription (qRT)-PCR to compare the levels of expression of pyruvate-metabolizing genes (Table S1) at 26°C (optimum) and 35°C (heat treatment). Ten minutes of heat treatment (to induce rapid-onset pyruvate accumulation) did not change the expression of glycolytic pathway genes that produce pyruvate but significantly downregulated two genes related to pyruvate consumption, those for pyruvate carboxylase and the tricarboxylic acid (TCA) cycle isocitrate dehydrogenase (Fig. 1B). Three hours of heat treatment (second upturn in pyruvate accumulation) downregulated additional genes related to pyruvate consumption encoding the respiratory components mitochondrial pyruvate permease, the β subunit in the pyruvate dehydrogenase complex that coverts pyruvate into acetyl coenzyme A (acetyl-CoA), and three rate-limiting enzymes in the TCA cycle (isocitrate dehydrogenase [α-ketoglutarate production], malate dehydrogenase [oxaloacetate production], and citrate synthase). Also downregulated were a lactate dehydrogenase that converts pyruvate into lactate (fermentation), an isocitrate lyase in the glyoxylate cycle, and a subunit of the acetolactate synthase that catalyzes the first step of amino acid (Ile, Leu, and Val) synthesis with pyruvate as the substrate. Heat treatment for 3 h upregulated the pyruvate kinase that catalyzes the production of pyruvate from phosphoenolpyruvate (Fig. 1B). Mechanisms of heat-induced pyruvate accumulation are summarized in Fig. 1C.

10.1128/mBio.01284-17.10 TABLE S1  Genes analyzed by qRT-PCR in this study. Download TABLE S1, PDF file, 0.1 MB. Copyright © 2017 Zhang et al. 2017 Zhang et al. This content is distributed under the terms of the Creative Commons Attribution 4.0 International license.

### Pyruvate scavenges heat-induced ROS.

We then investigated whether pyruvate accumulating in heat-stressed hyphae has a function. Exogenous administration of pyruvate can reduce ROS production in plants by activating the alternative respiratory pathway (12), but pyruvate does not activate this pathway in fungi (13). Pyruvate can protect mammalian cells by scavenging ROS; the α-keto-carboxylate structure in pyruvate neutralizes peroxides by reducing them to their conjugate alcohols, with the pyruvate being decomposed to acetate and CO_2_ (10). We first confirmed that acetate was produced nonenzymatically from pyruvate by mixing pyruvate and H_2_O_2_ in a test tube (Fig. S3). We then demonstrated that within 10 min of hyphae being transferred to 35°C, the intracellular acetate level increased 2-fold (*F* = 10.24, *P* = 0.011), reaching a plateau in 20 min (Fig. S3), consistent with intracellular pyruvate reacting with ROS.

10.1128/mBio.01284-17.3 FIG S3  *In vitro* assays of the abilities of α-keto acids to scavenge H_2_O_2_ in nonenzymatic reactions and intracellular accumulation of acetate in heat-treated hyphae. (A) Quantification of ROS in reaction mixtures containing H_2_O_2_ and an α-keto acid or glyceraldehyde. (B) Quantification of acetate in a reaction mixture containing H_2_O_2_ and pyruvate. (C) Accumulation of acetate in heat-stressed (35°C) hyphae. Values with different letters are significantly different (one-way ANOVA, *P* < 0.05). Download FIG S3, TIF file, 0.1 MB. Copyright © 2017 Zhang et al. 2017 Zhang et al. This content is distributed under the terms of the Creative Commons Attribution 4.0 International license.

**FIG 2:**
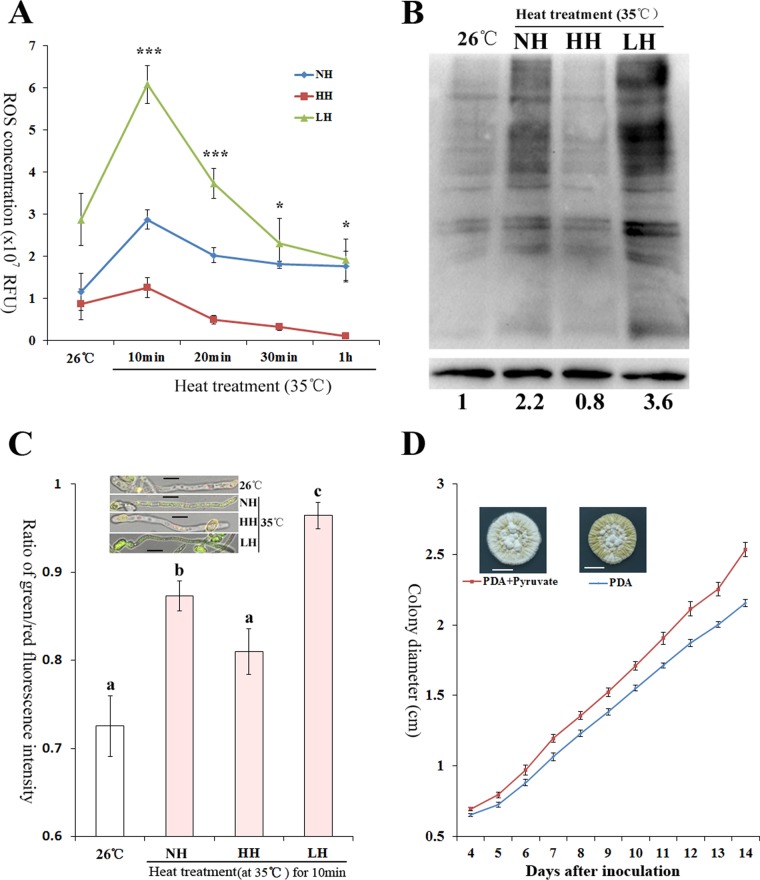
Pyruvate scavenges heat-induced ROS and protects fungal cells. (A) ROS levels in normal heat-treated hyphae (NH) and in hyphae with a higher (HH) or a lower (LH) pyruvate concentration than NH. ***, significant differences among NH, HH, and LH. *, ROS level in NH not different from that in LH but significantly higher than that in HH. RFU, relative fluorescence units. (B) Carbonylation level of total proteins in NH, LH, and HH (top) and protein level of β-tubulin (bottom). Presented are typical pictures representative of five repeats. Values indicate band intensities of total protein relative to β-tubulin; the value at 26°C was set to 1, and the other values were adjusted accordingly. (C) Pyruvate prevents the collapse of ΔΨm. The green/red fluorescence intensity ratio represents the extent to which the ΔΨm collapses. Presented are typical pictures representative of five repeats. Bars, 5 μm. Values with different letters are statistically significantly different (one-way ANOVA, *P* < 0.05). (D) Pyruvate (0.5 mM) promotes fungal growth on PDA plates under heat stress (35°C). The growth rates differed significantly (*t* test, *P* < 0.01) at ≥4 days postinoculation. Bars, 10 mm. Pictures were taken 14 days postinoculation. Presented are typical pictures representative of three repeats.

To confirm a role for pyruvate in the scavenging of heat-induced ROS, we produced heat-treated hyphae with different pyruvate concentrations and compared their ROS levels. Controls with normal levels of pyruvate (normal hyphae [NH]) were produced by mycelial growth at 26°C, followed by heat treatment. Hyphae with elevated (designated HH) or reduced (designated LH) pyruvate levels were prepared in the same way as NH but with the addition to the growth medium of pyruvate or a pyruvate kinase inhibitor, respectively (Fig. S4). At 10 min after heat treatment, HH and LH contained 395.7 ± 10.81 and 187.6 ± 9.70 μg of pyruvate/g of dried hyphae, respectively, compared to 261.6 ± 20.17 μg of pyruvate/g of dried hyphae in the NH control. We first determined if altering the pyruvate concentration impacts the expression of any of the previously identified ROS scavengers reviewed in reference 5. A genome-wide search identified seven SODs, nine catalases, one peroxiredoxin, and one glutathione peroxidase in *M. robertsii* (Table S1). The levels at which these ROS scavengers were expressed in NH, LH, and HH were similar (*P* > 0.05) (Fig. S4), indicating that pyruvate itself does not regulate the production of other scavengers. The ROS levels in NH, HH, and LH were also similar (*P* > 0.05) prior to heat treatment (Fig. 2A). Heat stress (35°C) induced a rapid spike in ROS levels in NH, which peaked at 10 min and by 20 min declined to the baseline level of non-heat-treated hyphae. Heat stress did not impact HH ROS levels, suggesting that the extra pyruvate completely blocked the ROS spike. Conversely, relative to the ROS levels in NH and HH, those in heat-stressed (10 min) LH rose 2.1-fold (*F* = 51.75, *P* = 2.8e^−4^) and 4.8-fold (*F* = 51.75, *P* = 2.6e^−4^), respectively (Fig. 2A). After 1 h at 35°C, NH and LH had similar ROS levels (*P* = 0.49), and they were significantly higher than those of HH (*F* = 7.32, *P* < 0.05).

10.1128/mBio.01284-17.4 FIG S4  Preparation of heat-stressed hyphae with different pyruvate concentrations (A) and their impacts on the expression levels of ROS scavengers (nine catalases, seven SODs, one peroxiredoxin, and one glutathione peroxidase) (B). NH, normal heat-stressed hyphae. To prepare NH, hyphae grown at 26°C for 36 h in 1/2SDY were subjected to heat treatment (35°C). HH, hyphae with significantly higher pyruvate concentration than NH. To prepare HH, hyphae grown at 26°C for 36 h in 1/2SDY plus 6 mM pyruvate were subjected to heat treatment. LH, hyphae with significantly lower pyruvate concentration than NH. To prepare LH, hyphae were grown at 26°C for 36 h in 1/2SDY, treated with pyruvate kinase inhibitor (4 μg/ml) for 1 h, and heat treated. *, Significant difference (one-way ANOVA, *P* < 0.05). Note that, as shown in panel B, there were no significant differences (one-way ANOVA, *P* > 0.05) among NH, LH, and HH in the expression levels of the genes listed. Download FIG S4, TIF file, 0.2 MB. Copyright © 2017 Zhang et al. 2017 Zhang et al. This content is distributed under the terms of the Creative Commons Attribution 4.0 International license.

### Accumulated pyruvate protects fungal cells from ROS damage.

ROS can carbonylate amino acid side groups, and the resulting carbonylated proteins form aggregates (14). Heat treatment increased the level of protein carbonylation in NH relative to that in HH, whereas LH had the highest protein carbonylation level (Fig. 2B). ROS also destabilizes the mitochondrial cell membrane potential (ΔΨm) (15). Heat treatment induced a significantly (*F* = 41.54, *P* = 2.05e^−7^) greater ΔΨm collapse in NH than in HH, and the ΔΨm collapse was most pronounced in LH (*F* = 41.54, *P* < 0.01) (Fig. 2C). Moreover, addition of pyruvate (0.5 mM) to potato dextrose agar (PDA) had no impact on fungal growth at the optimal temperature (26°C) (Fig. S5) but significantly (one-way analysis of variance [ANOVA], *P* < 0.05) enhanced growth under heat stress (35°C) (Fig. 2D). There are two other α-keto acids (oxaloacetate and α-ketoglutarate) in the TCA cycle; the addition of either oxaloacetate or α-ketoglutarate also promoted fungal growth under heat stress (Fig. S5), consistent with the ability to scavenge ROS (Fig. S3). However, the intracellular level of α-ketoglutarate decreased ~6-fold (*F* = 49.40, *P* = 3.6e^−7^) and oxaloacetate became undetectable 10 min following a transfer to 35°C (Fig. S6). This was concurrent with the downregulation of the genes for their production (Fig. 1B). Therefore, pyruvate is the only upregulated α-keto acid that is able to scavenge heat-induced ROS. As an additional control, we tested glyceraldehyde (not a α-keto acid) and confirmed that it had no impact on fungal growth under heat stress (Fig. S5) and that it is not able to scavenge ROS (Fig. S3).

10.1128/mBio.01284-17.5 FIG S5  Growth curves of *M. robertsii* on PDA plates with or without an α-keto acid or glyceraldehyde at the optimal temperature (26°C) or under heat stress (35°C). Growth curves on PDA plates supplemented with a chemical at 26°C (A, oxaloacetate; C, α-ketoglutarate; E, glyceraldehyde; G, pyruvate) and 35°C (B, oxaloacetate; D, α-ketoglutarate; F, glyceraldehyde). At 35°C, significant differences between PDA and PDA supplemented with oxaloacetate or α-ketoglutarate were seen at >4 days postinoculation (*t* test, *P* < 0.05). Download FIG S5, JPG file, 0.6 MB. Copyright © 2017 Zhang et al. 2017 Zhang et al. This content is distributed under the terms of the Creative Commons Attribution 4.0 International license.

10.1128/mBio.01284-17.6 FIG S6  Impacts of heat treatment on two α-keto acids, expression of genes encoding HSPs and components of the TRX/TRR-GRX/GLR system, and trehalose. Panels: A, α-ketoglutarate; B, oxaloacetate. In panels A and B, an asterisk indicates a significant difference between the hyphae grown at 26 and 35°C (*t* test, *P* < 0.01). Note that oxaloacetate was not detectable in heat-treated hyphae. (C) qRT-PCR analysis of genes encoding HSPs and components of the TRX/TRR-GRX/GLR system in response to heat treatment. The expression level of a gene at 26°C is set to 1; the values represent the relative expression of the gene after different time periods of heat treatment versus that at 26°C. (D) Trehalose production in hyphae at 26°C and under heat stress (35°C) for different time periods. Values with different letters are significantly different (one-way ANOVA, *P* < 0.05). Download FIG S6, JPG file, 0.6 MB. Copyright © 2017 Zhang et al. 2017 Zhang et al. This content is distributed under the terms of the Creative Commons Attribution 4.0 International license.

### Pyruvate accumulates before other ROS scavengers in heat-treated hyphae and conidia.

**FIG 3:**
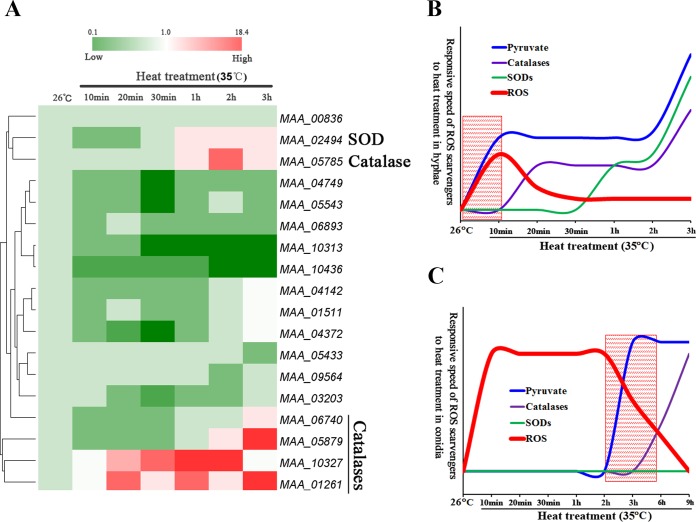
Pyruvate is in the vanguard of molecules that scavenge heat-induced ROS. (A) qRT-PCR analysis of expression of ROS scavengers (catalases, SODs, peroxiredoxin, and glutathione peroxidase) in response to heat treatment. The expression level of a gene at 26°C is set to 1; the values represent the relative expression of the gene after different time periods of heat treatment versus that at 26°C. (B) Diagrams showing the relative speeds of response of ROS scavengers to heat treatment in hyphae according to the data presented in Fig. 1A and 2A. The shadowed area shows where pyruvate is the only scavenger responding to rapid induction of ROS (the first 10 min). (C) Diagrams showing the relative speeds of response of ROS scavengers to heat treatment in conidia based on the data presented in Fig. S7. The shadowed area demonstrates that pyruvate is the first ROS scavenger responding to heat treatment.

We then investigated the relationship between pyruvate accumulation and the expression of other cellular protective responses to heat stress (Table S1). Only four catalases and one SOD were upregulated upon heat treatment, and all after the increase in pyruvate (Fig. 3A and B). RNA-Seq analyses also identified the upregulation of three small HSP-encoding genes and five genes in the TRX-TRR and GRX-GLR systems (Fig. S2A); qRT-PCR showed that a small HSP gene (MAA_10381) was upregulated ~4-fold (*t* = 6.52, *P* = 0.02) 10 min after heat treatment, and the other genes were upregulated at various subsequent time points (Fig. S6). Synthesis of trehalose was significantly (*F* = 9.84, *P* = 0.008) increased after 30 min of heat treatment and peaked at 9 h (Fig. S6).

10.1128/mBio.01284-17.7 FIG S7  Responses of *M. robertsii* conidia to heat stress. (A) ROS levels in heat-treated conidia (35°C). *, Significantly different from non-heat-treated conidia (one-way ANOVA, *P* < 0.05). (B) Production of pyruvate in conidia grown at the optimal temperature (26°C) and 35°C. (C) Trehalose concentrations in hyphae and conidia incubated at 26°C (empty columns) and 35°C (red columns). In panels B and C, values with different letters are significantly different (one-way ANOVA, *P* < 0.05). qRT-PCR was used to measure the expression of genes encoding HSPs and components of the TRX/TRR-GRX/GLR system (D) and ROS scavengers (catalases, SODs, a peroxiredoxin, and a glutathione peroxidase) (E) at 26 and 35°C. The expression level of a gene at 26°C is set to 1; the values represent the levels of expression of the gene after different periods of heat treatment relative to that at 26°C. Download FIG S7, TIF file, 0.3 MB. Copyright © 2017 Zhang et al. 2017 Zhang et al. This content is distributed under the terms of the Creative Commons Attribution 4.0 International license.

Most fungi use asexually produced conidia as a stress-resistant dispersal stage. As in hyphae, heat treatment (35°C) induced rapid ROS production in dormant conidia (Fig. S7). This peaked at 10 min (*F* = 13.76, *P* = 0.013) and stayed level for 3 h before declining at the same time as a 1.3-fold increase (*t* = 5.24, *P* = 0.03) in the pyruvate concentration (Fig. 3C; Fig. S7). Two small HSPs were upregulated ~8-fold (*P* < 0.05) in conidia after 10 min at 35°C (Fig. S7), and two catalases were upregulated (*P* < 0.05) after 6 h (Fig. 3C; Fig. S7). The conidial trehalose concentration was not altered by heat treatment; however, conidia harvested from cultures grown at 26°C already contained a higher trehalose concentration than hyphae (Fig. S7).

### Hog1-MAPK regulates tolerance to heat-induced acid stress.

**FIG 4:**
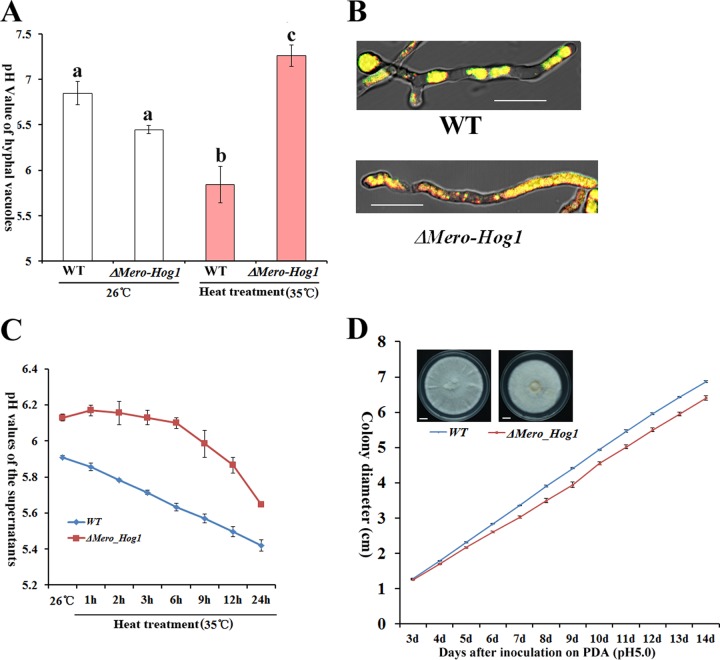
Hog1-MAPK is involved in tolerance to heat-induced acid stress in *M. robertsii*. (A) Vacuolar pH values in hyphae grown at the optimal temperature and after 10 min at 35°C. Values with different letters are significantly different (one-way ANOVA, *P* < 0.05). (B) Heat-treated hyphae (35°C for 10 min) stained with BCECF-AM. Note that more red spots are visible in the Hog1-MAPK (Δ*Mero-Hog1*) deletion mutant, suggesting a pH higher than that of the WT strain. Bars, 10 μm. Presented are typical pictures representative of five repeats. (C) pH values in supernatants of heat-treated WT and Δ*Mero-Hog1* mutant cultures. (D) Growth curves of WT and Δ*Mero-Hog1* mutant hyphae on buffered PDA plates (pH 5) at the optimal temperature (26°C). The growth rates differed significantly (*t* test, *P* < 0.01) at ≥4 days postinoculation. Bars, 10 mm. Pictures were taken 14 days postinoculation. Presented are typical pictures representative of three repeats.

As described above, scavenging of ROS by pyruvate produces acetate, which is known to cause acid stress in other fungi (16). We indeed found that both intracellular vacuoles and culture supernatant of *M. robertsii* were acidified upon heat treatment (Fig. 4A to C), suggesting that the heat-treated hyphae were under acid stress. *Saccharomyces cerevisiae* tolerates acetate stress in a Hog1 mitogen-activated protein kinase (MAPK)-regulated process that involves the translocation of protons into vacuoles and out of cells (16). Hog1-MAPK is involved in tolerance to several abiotic stresses in *M. robertsii* (17); in this study we found that the growth of a Hog1-MAPK (Δ*Mero-Hog1*) deletion mutant was also dramatically inhibited under heat stress (Fig. S8). Heat treatment did not change the pyruvate concentration and ROS levels in the Δ*Mero-Hog1* mutant relative to those in the wild-type (WT) strain (Fig. S8), but the pH values of culture supernatants (*t* = 7.19, *P* = 0.01) and vacuoles (*t* = 3.47, *P* = 0.02) were significantly higher (Fig. 4A to C). These data suggest that the heat-treated Δ*Mero-Hog1* mutant could be impaired in the translocation of protons into the vacuoles and out of cells. Like the growth of the WT, that of the Δ*Mero-Hog1* mutant was also inhibited by acid stress at 26°C (Fig. 4D), indicating that Hog1-MAPK is also involved in acid stress tolerance at the optimal temperature. Therefore, *M. robertsii* uses a conserved regulatory mechanism to tolerate heat-induced acid stress.

10.1128/mBio.01284-17.8 FIG S8  Involvement of Hog1-MAPK in heat stress tolerance. (A) Growth curves of the WT and the *Hog1-MAPK* (Δ*Mero-Hog1*) deletion mutant on PDA plates at 26°C. (B) Pictures of WT and Δ*Mero-Hog1* mutant colonies on PDA plates taken at 14 days postinoculation. Bars, 10 mm. (C) Pyruvate concentrations in WT and Δ*Mero-Hog1* mutant hyphae grown in 1/2SDY at 26 or 35°C for 3 h. Values with different letters are significantly different (one-way ANOVA, *P* < 0.05). (D) ROS concentrations in heat-stressed (35°C for 10 min) WT and Δ*Mero-Hog1* mutant hyphae. Values with different letters are significantly different (*t* test, *P* < 0.05). Download FIG S8, TIF file, 0.5 MB. Copyright © 2017 Zhang et al. 2017 Zhang et al. This content is distributed under the terms of the Creative Commons Attribution 4.0 International license.

### Heat treatment induces pyruvate accumulation in five other fungi.

We investigated whether heat treatment also induces pyruvate accumulation in five other fungi. *Magnaporthe oryzae* is similar to *M. robertsii* in heat tolerance (18), whereas *Neurospora crassa*, *Aspergillus fumigatus*, and *S. cerevisiae* can grow at temperatures above 42°C (18–20) and *Cordyceps militaris*, with an optimal temperature of ~20°C (21), does not grow at 30°C (Fig. S9). Heat treatment induced pyruvate accumulation in all five fungi. Similar to the pyruvate concentration in *M. robertsii*, that in *M. oryzae* and *N. crassa* was increased ~2-fold (one-way ANOVA, *P* < 0.05) after 10 min of heat treatment, that in *A. fumigatus* and *C. militaris* was significantly elevated (one-way ANOVA, *P* < 0.05) after 20 min, and that in *S. cerevisiae* was significantly elevated after 30 min (Fig. S9).

10.1128/mBio.01284-17.9 FIG S9  Heat-induced pyruvate accumulation in five fungi, *N. crassa* (A), *A. fumigatus* (B), *S. cerevisiae* (C), *M. oryzae* (D), and *C. militaris* (E). *, Significant differences between pyruvate concentrations in hyphae grown at the respective optimal temperature and after heat treatment (*P* < 0.05). (F) Growth of *C. militaris* on PDA plates at different temperatures. The pictures were taken at 10 days postinoculation. Bars, 10 mm. Note that no observable growth occurred at 30°C. Download FIG S9, JPG file, 0.9 MB. Copyright © 2017 Zhang et al. 2017 Zhang et al. This content is distributed under the terms of the Creative Commons Attribution 4.0 International license.

## DISCUSSION

In this study, we provide the first evidence that heat stress induces pyruvate accumulation in a fungus and that it efficiently scavenges heat-induced ROS. This results in a reduction of protein carbonylation, stabilization of the ΔΨm, and promotion of fungal growth. A very notable feature of pyruvate accumulation in *M. robertsii* hyphae is the rapidity of its induction, coincident with ROS production, and its speed of action as a scavenger. Other ROS scavengers (catalases and SODs) are induced by heat stress later than pyruvate, suggesting that pyruvate plays a dominant role in scavenging rapid-onset ROS and pyruvate subsequently works with other scavengers to maintain low levels of ROS. Moreover, we found that pyruvate is also the first ROS scavenger produced by conidia in response to heat treatment. Our study identified changes in the expression levels of the pyruvate metabolism genes as a major contributor to the accumulation of pyruvate, but other mechanisms could also be involved. Thus, the activities of pyruvate-metabolizing enzymes could be altered by heat treatment, which could also contribute to pyruvate accumulation.

Along with pyruvate accumulation in response to heat treatment, hyphae and conidia rapidly produce one and two small HSPs, respectively, that presumably protect proteins from degradation. Cyclophilins respond quickly to heat stress in other fungi such as *Beauveria bassiana* (22). Although they were not identified by our RNA-Seq analysis, if these proteins were constitutively expressed or stored in the cell, they could also be involved in the *M. robertsii* response to heat stress.

Previous studies have not identified pyruvate accumulations as a heat stress tolerance mechanism in fungi either because they generally focused on the roles of genes that increase in response to heat stress or screened nonessential gene mutants with altered heat stress tolerance (3, 5). We instead looked at genes that either were not changed or were downregulated by heat treatment and are involved in key metabolic pathways, e.g., pyruvate metabolism. Pyruvate accumulates after a short (10-min) heat treatment because of downregulation of genes for pyruvate consumption. Prolonged heat treatment downregulates additional genes involved in pyruvate consumption and also upregulates the gene for pyruvate kinase, the last enzyme in the glycolytic pathway for pyruvate production, accelerating pyruvate accumulation. Similarly, mammalian cells respond to hypoxia by increasing the production of glycolytic enzymes and reducing pyruvate consumption (e.g., TCA cycle and neoglucogenesis enzymes) (10). Presumably, this functions principally to increase ATP availability despite a decreased oxygen supply. Nevertheless, conservation of these processes for stress-induced pyruvate accumulation in mammalian cells and *M. robertsii* suggests that heat-induced pyruvate accumulation in other eukaryotic cells will proceed by similar mechanisms.

Pyruvate has metabolic and redox properties and is located at a key intersection in the network of metabolic pathways (10). Although the addition of 0.5 mM pyruvate to cultures promoted the growth of heat-stressed *M. robertsii*, the addition of more pyruvate (2 mM) is harmful to *S. cerevisiae* (11). This implies that the 1.5-fold increase in pyruvate induced by heat stress is a balanced response to ROS scavenging and the need to maintain metabolic homeostasis.

In addition to *M. robertsii*, we found that heat treatment also induces pyruvate accumulation in five phylogenetically distant fungi that differ in the ability to tolerate heat stress, suggesting that scavenging of heat-induced ROS by pyruvate is widespread in fungi. Discovering how *Metarhizium* species responds to environmental stress has particular significance for the development of these fungi as environmentally friendly alternatives to chemical insecticides in biocontrol programs (23, 24). Currently, mycoinsecticides have a small market share, in part because of susceptibility to heat stress and UV radiation (25). The novel mechanisms of heat stress tolerance we describe could serve as a theoretical base for the improvement of mycoinsecticides.

## MATERIALS AND METHODS

### Fungal strains.

*M. robertsii* ARSEF2575 and *C. militaris* ARSEF4081 were obtained from the Agricultural Research Service Collection of Entomopathogenic Fungi, *N. crassa* FGSC 4200 and *A. fumigatus* Af293 were from the Fungal Genetics Stock Center, and *S. cerevisiae* CICC1308 was from the China Center of Industrial Culture Collection. *M. oryzae* P131 was a gift from Youliang Peng at the China Agricultural University.

### Transcriptomic analysis by RNA-Seq.

RNA-Seq was used to compare the transcriptomes of hyphae grown at the optimal temperature (26°C) and those heat stressed at 35°C for 3 h. To achieve this, mycelial cultures were prepared by inoculating 1 × 10^8^ *M. robertsii* conidia into 100 ml of 1/2SDY and shaking it (200 rpm) for 36 h at 26°C. Cultures were then incubated for 3 h with shaking at either 26°C (optimal temperature) or 35°C (heat stress). Mycelium was harvested for RNA extraction with TRIzol reagent (Life Technologies, Inc.). Construction of libraries and sequencing with the Illumina HiSeq 2500 platform were performed by Woosen Biotechnology Co. Ltd. (Hangzhou, China). After paired-end sequencing, clean reads were obtained with the NGS QC Toolkit and then mapped to the draft genome sequence of *M. robertsii* (26) with the program TopHat 2.0.6 (27). Reads that aligned uniquely with the reference sequence were used for gene expression quantification by the RPKM (reads per kilobase per million reads) method (28). Differential expression analysis was performed with DESeq software (29) by using cutoffs of an adjusted *P* value of 0.05 and a ≥2-fold change (Benjamini-Hochberg method). Two biological replicates were established for each treatment. The pathway enrichment analysis of DEGs was based on the KEGG database.

### qRT-PCR analysis.

qRT-PCR was conducted as previously described (16). Two genes (*act*, *tef*) were used as internal standards (30). The relative normalized transcript level of each gene was computed by the 2^−ΔΔ*CT*^ method (31). The primers used in qRT-PCR are listed in Table S1. cDNA was synthesized with ReverTra Ace qPCR RT master mix by using gDNA remover (Toyobo, Japan). Quantitative PCR was conducted with Thunderbird SYBR qPCR mix (no ROX reference dye; Toyobo, Japan). Each treatment had three replicates, and the experiments were repeated three times.

### Time course of heat treatment of *M. robertsii* hyphae and conidia.

Hyphal cultures grown at 26°C for 36 h were prepared as described above, aliquoted into 50-ml tubes (10-ml culture volume in each tube), and heat treated at 35°C for 10, 20, or 30 min or 1, 2, 3, 6, 9, 12, or 24 h. The heat-treated hyphae were collected for RNA isolation. Hyphae were lyophilized on a freeze dryer (LABCOTT) for quantification of pyruvate, acetate, oxaloacetate, α-ketoglutarate, ROS, and trehalose (described below). To obtain conidia for heat treatment, 100 μl of a conidial suspension (1 × 10^6^ conidia/ml) was evenly spread onto PDA plates (BD), which were then incubated at 26°C for 15 days to produce mature conidia. The plates were then incubated at 35°C for 10, 20, or 30 min or 1, 2, 3, 6, 9, 12, or 24 h. After heat treatment, conidia were immediately suspended in 0.01% (vol/vol) Triton X-100 and equal numbers of conidia were used for the isolation of RNA or quantification of pyruvate, acetate, ROS, or trehalose. All experiments were repeated three times.

### Quantification of three α-keto acids (pyruvate, oxaloacetate, α-ketoglutarate), trehalose, ROS, and acetate.

To quantify α-keto acids, ROS, trehalose, and acetate, lyophilized hyphae (10 mg) were placed in 0.4 ml of phosphate-buffered saline (PBS; 50 mM, pH 7.4) in a 1.5-ml Eppendorf tube with ceramic beads (diameter, 0.3 mm), and homogenized in a grinder (Jinxing, Shanghai, China). The supernatants obtained after 10 min of centrifugation (12,000 × *g*) at 4°C were used for subsequent assays. The pyruvate and α-ketoglutarate in the supernatant (50 μl) was quantified with a pyruvate assay kit (Abcam, Inc.) and an α-ketoglutarate assay kit (Sigma-Aldrich), respectively. For oxaloacetate quantification, the supernatant was concentrated 2-fold by lyophilization and subjected to a fluorometric assay with an oxaloacetate colorimetric/fluorometric assay kit (BioVision). For total ROS quantification, the supernatant was diluted 500-fold with PBS buffer and 50 μl of the diluted supernatant was subjected to ROS quantification with the OxiSelect *In Vitro* ROS/RNS assay kit (Cell Biolabs, Inc.). To quantify trehalose, the supernatant was diluted 11-fold in PBS buffer and 60 μl of the diluted supernatant was assayed with a trehalose quantification kit (Cablebridge Biotechnology Co., Ltd., Shanghai, China). For acetate quantification, 6.25 μl of the supernatant was assayed with an acetate quantification kit (Roche, Germany), which is an acetyl-CoA synthetase-based kit that is not influenced by pyruvate in the samples tested.

To quantify pyruvate and ROS in conidia, ~10^8^ conidial cells were treated with liquid nitrogen, which killed all of the conidia (a sample showed zero germination in 1/2SDY at 26°C). The liquid nitrogen-treated conidia were incubated at 26°C for 2 h in 0.01% Zymolyase (Seikagaku, Biobusiness, Japan) and 10% snailase (Solarbio, China) to partially remove cell walls. The treated conidia were collected and washed twice with PBS buffer, suspended in 400 μl of PBS, and homogenized with ceramic beads (diameter, 1 mm) in a grinder (Jinxing, Shanghai, China). The supernatant was then subjected to pyruvate, trehalose, and acetate quantification with the respective kits. All quantification assays were repeated three times with two replicates per repeat.

### Preparation of heat-treated hyphae with different pyruvate concentrations.

Normal heat-stressed (35°C) hyphae (designated NH) were prepared as described above. To prepare heat-treated hyphae with a higher pyruvate concentration than NH (designated HH), conidia were grown in 100 ml of 1/2SDY supplemented with pyruvate (6 mM). The hyphae were then aliquoted into 50-ml tubes (10 ml in each tube) and heat stressed in the same way as NH. To prepare heat-treated hyphae with a lower pyruvate concentration than NH (designated LH), conidia were grown in 1/2SDY at 26°C for 36 h; the culture was then treated with 4 μg/ml pyruvate kinase inhibitor (NCGC00188636; Sigma) at 26°C for 1 h and heat stressed.

### Assays of growth under heat stress and acid stress.

To assay the growth of *M. robertsii* under heat stress, 5 μl of a conidial suspension (10^7^ conidia/ml) was applied to the center of a PDA plate (diameter, 90 mm) that was then incubated at 26°C (optimal growth) or at 35 or 37°C (heat stress). Starting 4 days postinoculation, the colony diameter was measured daily.

To assay the effects of α-keto acids (pyruvate, oxaloacetate, α-ketoglutarate) and glyceraldehyde on the growth of *M. robertsii* under heat stress, the PDA plates were supplemented with pyruvate (0.5 mM), oxaloacetate (0.1 mM), α-ketoglutarate (0.5 mM), or glyceraldehyde (0.5 mM). The addition of these chemicals at the concentrations described to PDA plates had no impact on fungal growth at 26°C.

To assay the growth of *M. robertsii* under acid stress, the same conidial suspension was inoculated onto PDA plates buffered with 2-(*N*-morpholino)ethanesulfonic acid (MES) to pH 5.0. All growth assays were repeated three times with three replicates per repeat.

### Assay of protein carbonylation.

To assay protein carbonylation in *M. robertsii*, hyphae were ground into a fine powder in liquid nitrogen and the total protein was extracted overnight at 4°C in a mixture of 0.1 M Tris-HCl (pH 7.4), 0.15 M NaCl, 1 mM EDTA, 1% (vol/vol) Triton X-100, 0.5% SDS, and 2% β-mercaptoethanol plus a protease inhibitor cocktail (Sigma). The protein concentration was determined with the bicinchoninic acid protein quantitation assay kit (KeyGen Bio TECH, Nanjing, China). Twenty micrograms of the total protein was subjected to derivatization with 2,4-dinitrophenylhydrazine as described in the instructions for the OxyBlot protein oxidation detection kit (Millipore). The protein was then fractionated by 10% SDS-PAGE, and the gel was soaked in 1% β-mercaptoethanol in 1× gel running buffer. The proteins were electroblotted to a polyvinylidene difluoride membrane, and Western blot analysis was conducted to detect protein carbonylation with rabbit anti-dinitrophenol antibody in accordance with the manual for the OxyBlot protein oxidation detection kit (Millipore).

### Assays of ΔΨm.

The ΔΨm of hyphae was monitored by confocal microscopy with JC-1 (a ΔΨm-dependent dye) as previously described (32), with a JC-1 staining kit from Beyotime Biotechnology (Shanghai, China). Sixty microliters of each conidial suspension (4 × 10^7^ conidia/ml) was inoculated into 3 ml of 1/2SDY in a petri dish (Corning), incubated at 26°C for 15 h, and subjected to heat treatment for 10 min. The medium was removed, and the hyphae were rinsed twice with distilled water. The JG-1 staining solution was then applied for 30 min, and then the hyphae were rinsed three times with staining buffer. The fluorescence intensities at wavelengths of 490 (excitation) and 530 (emission) nm and at 525 (excitation) and 590 (emission) nm were measured by confocal microscopy (Zeiss, Germany), and their ratio was calculated with the ImageJ software (NCBI).

To prepare hyphae with an elevated pyruvate concentration (HH) for JG-1 staining, conidia were cultured on diagnostic slides for 15 h in 1/2SDY plus pyruvate (6 mM) before heat treatment. To prepare hyphae with a reduced pyruvate concentration (LH), conidia were cultured on diagnostic slides for 14 h. The regular 1/2SDY was then replaced with 1/2SDY containing the pyruvate kinase inhibitor (described above), and the slide was incubated for another 1 h at 26°C before heat treatment. The experiments were repeated five times with three replicates per repeat.

### Assays of vacuolar pH and extracellular pH.

Hyphal cultures growing on the hydrophobic surface of diagnostic slides were prepared as described above. Vacuolar pH values in the hyphae was assayed with 2′,7′-bis(2-carboxyethyl)-5(6)-carboxyfluorescein-acetoxymethyl ester (BCECF-AM) as previously described (33). Briefly, hyphae were rinsed three times with PBS buffer, stained with the BCECF-AM staining solution for 30 min, and then rinsed twice with PBS buffer. The fluorescence intensities at wavelengths of 450 (excitation) and 535 (emission) nm and at 490 (excitation) and 535 (emission) nm were measured by confocal microscopy. BCECF-AM was purchased from Beyotime Biotechnology (Shanghai, China)

The extracellular pH, i.e., the pH of the culture supernatant, was measured with a pH meter (Mettler Toledo, Switzerland). The experiments were repeated five times with three replicates per repeat.

### Statistical analysis.

Tukey’s honestly significant difference test in the OriginPro 8.5 program was used for statistical analysis of the data obtained in this study (OriginLab).

### Accession number(s).

The GenBank accession number for all of the RNA-Seq sequencing data obtained in this study is SRR5282608.
