# Supplementary figures and images for: Pyruvate Accumulation Is the First Line of Cell Defense against Heat Stress in a Fungus
Source: mBio. 2017 Sep 5;8(5):e01284-17. doi: 10.1128/mBio.01284-17 (PMC5587913; doi:10.1128/mBio.01284-17)

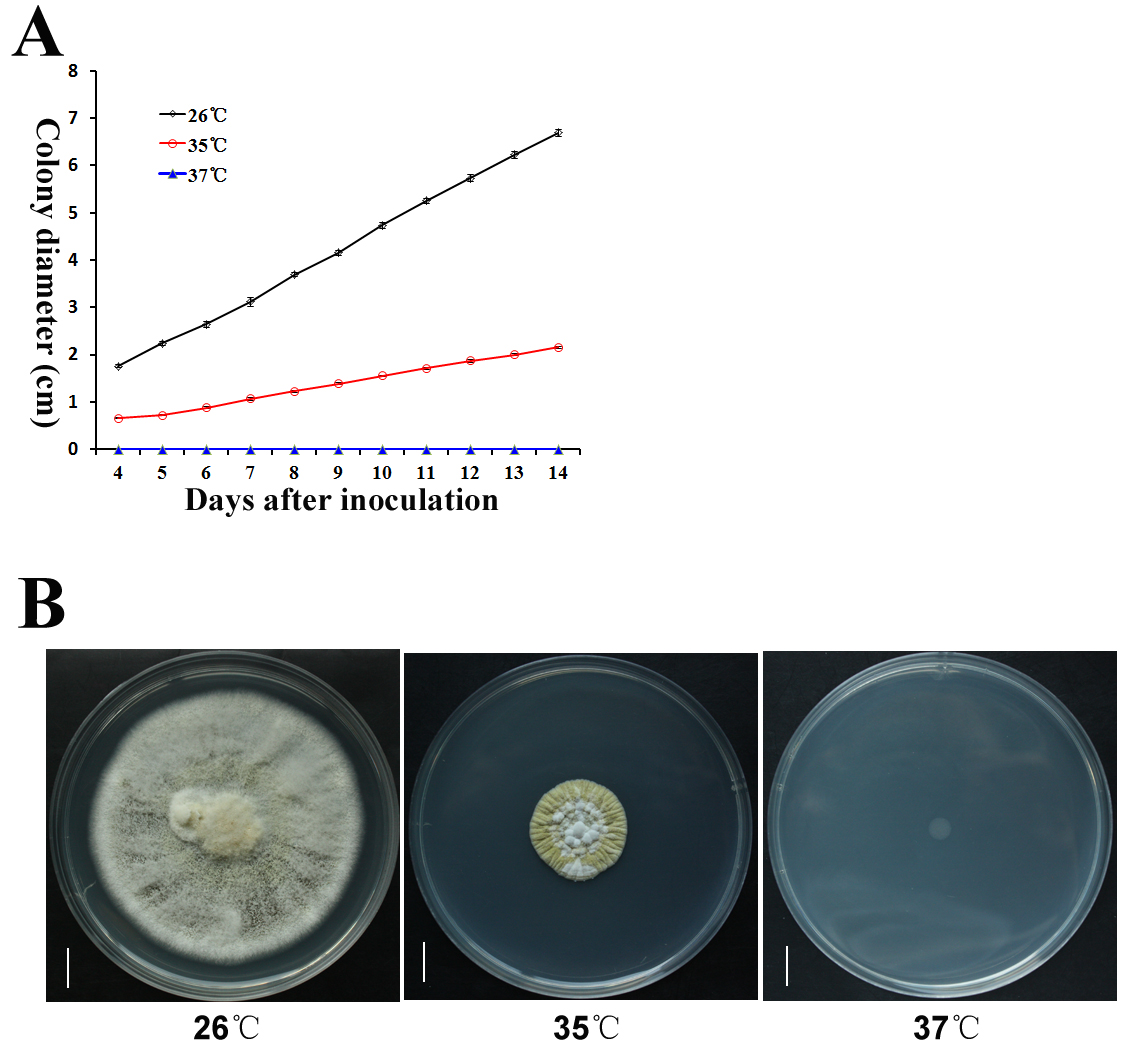

Supplement: FIG S1 [file mbo004173466sf1.jpg]

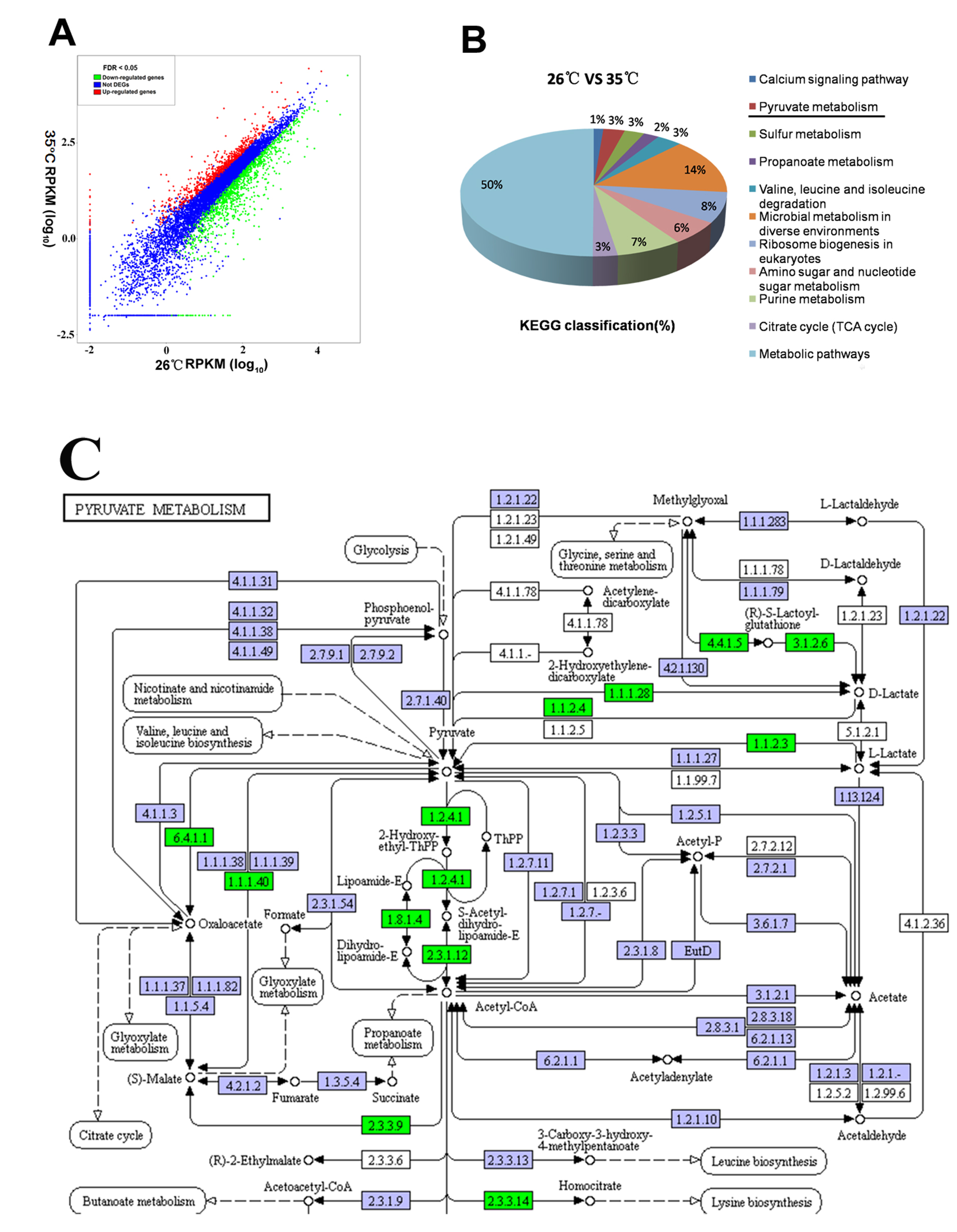

Supplement: FIG S2 [file mbo004173466sf2.jpg]

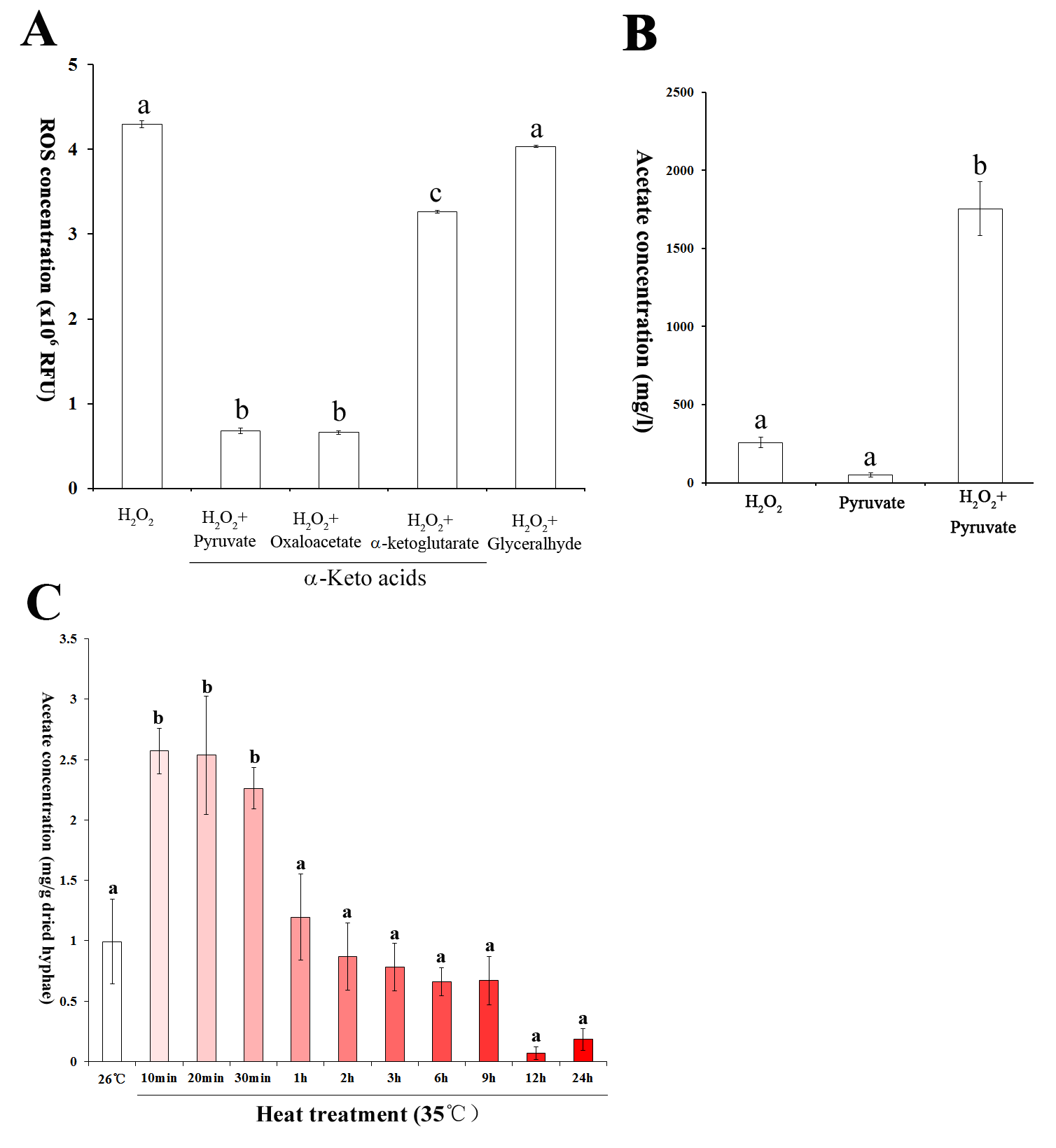

Supplement: FIG S3 [file mbo004173466sf3.tif]

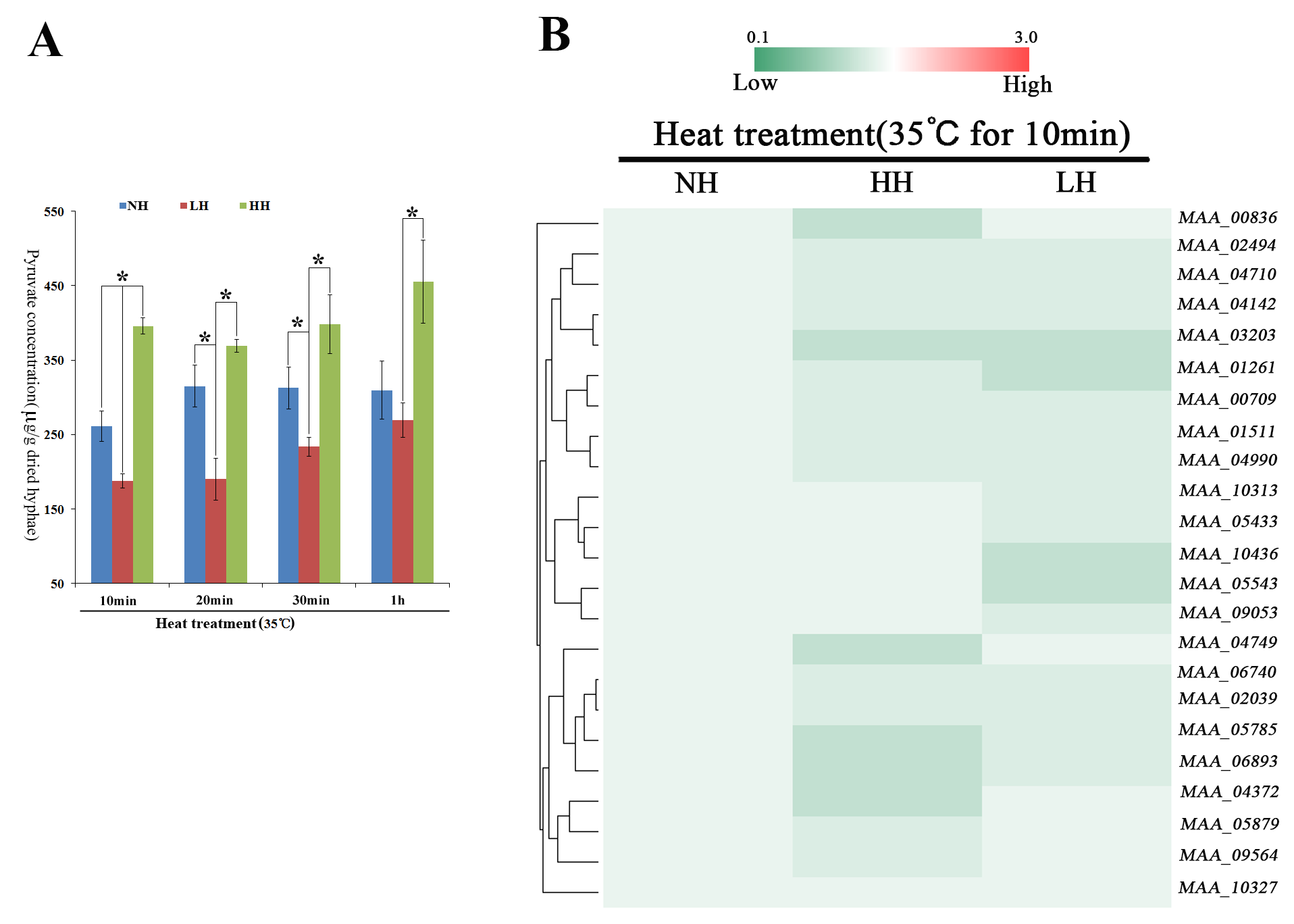

Supplement: FIG S4 [file mbo004173466sf4.tif]

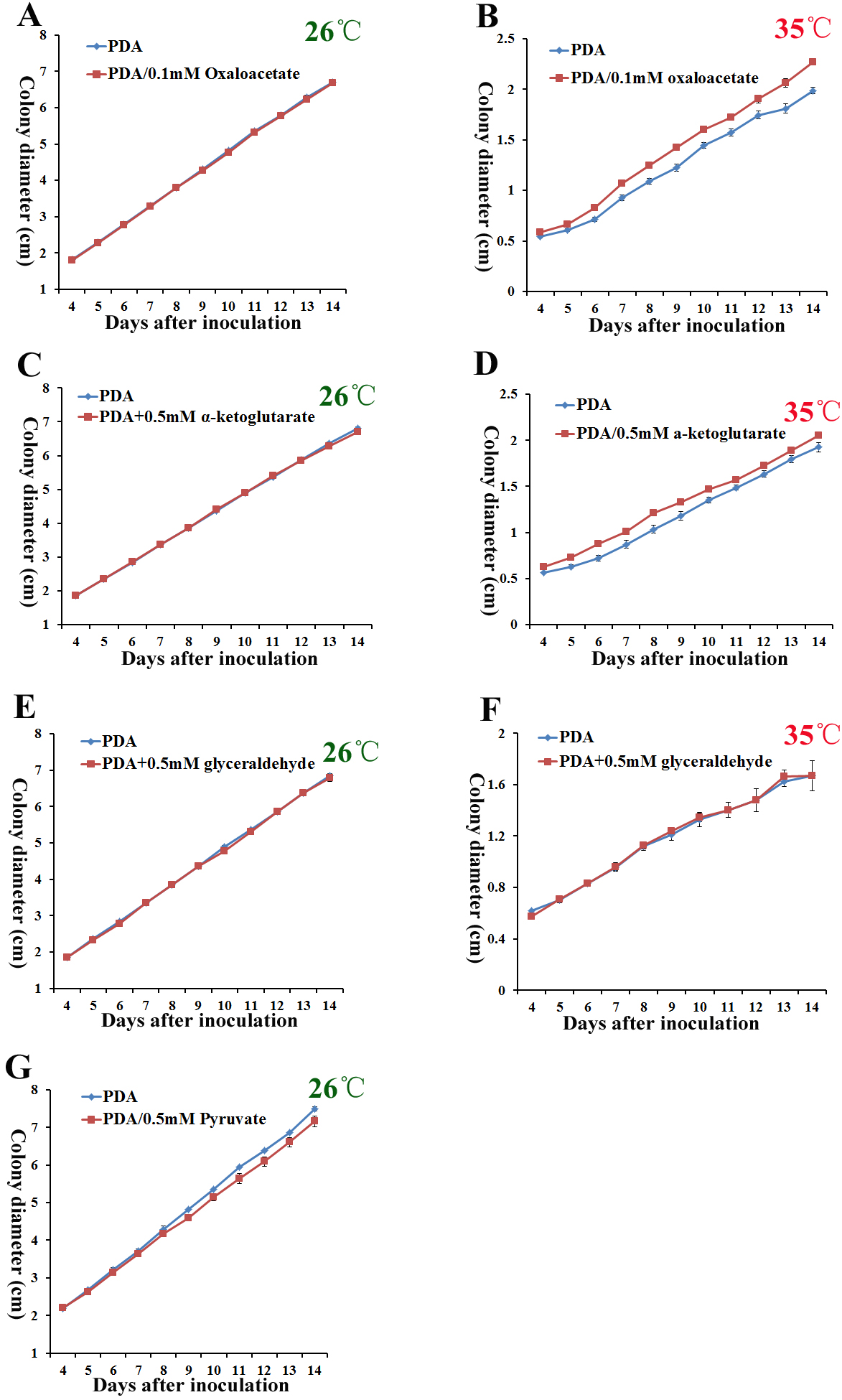

Supplement: FIG S5 [file mbo004173466sf5.jpg]

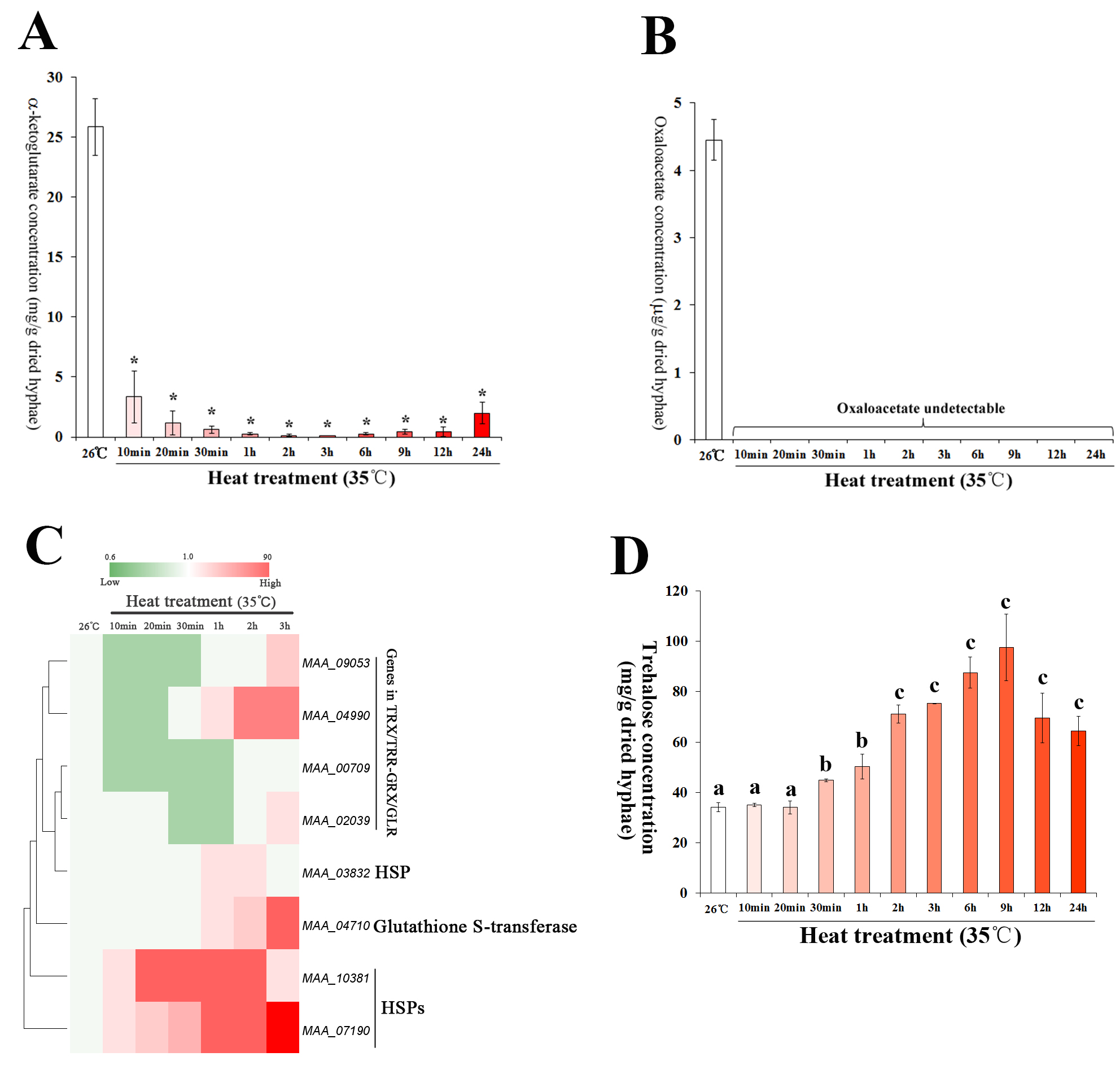

Supplement: FIG S6 [file mbo004173466sf6.jpg]

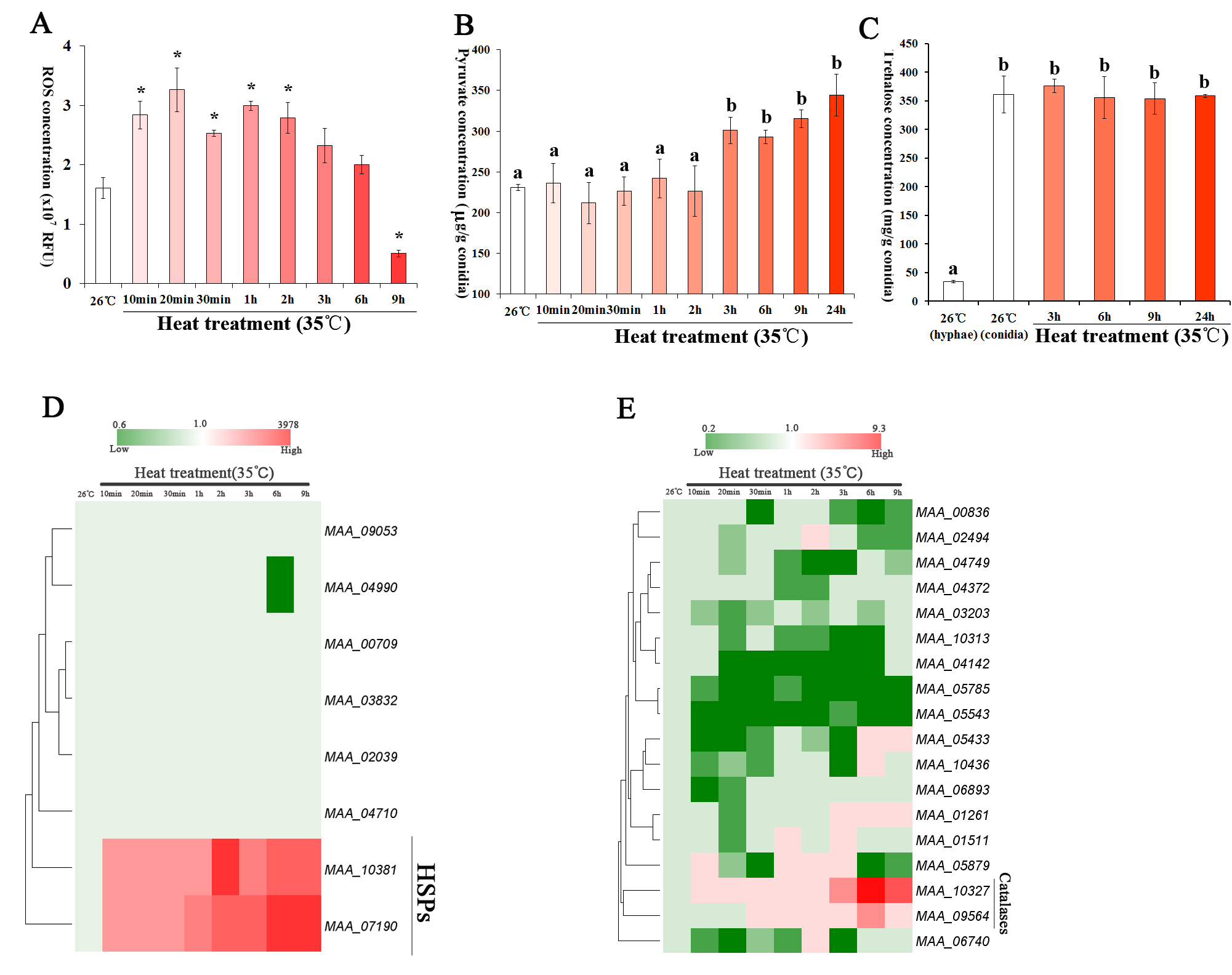

Supplement: FIG S7 [file mbo004173466sf7.tif]

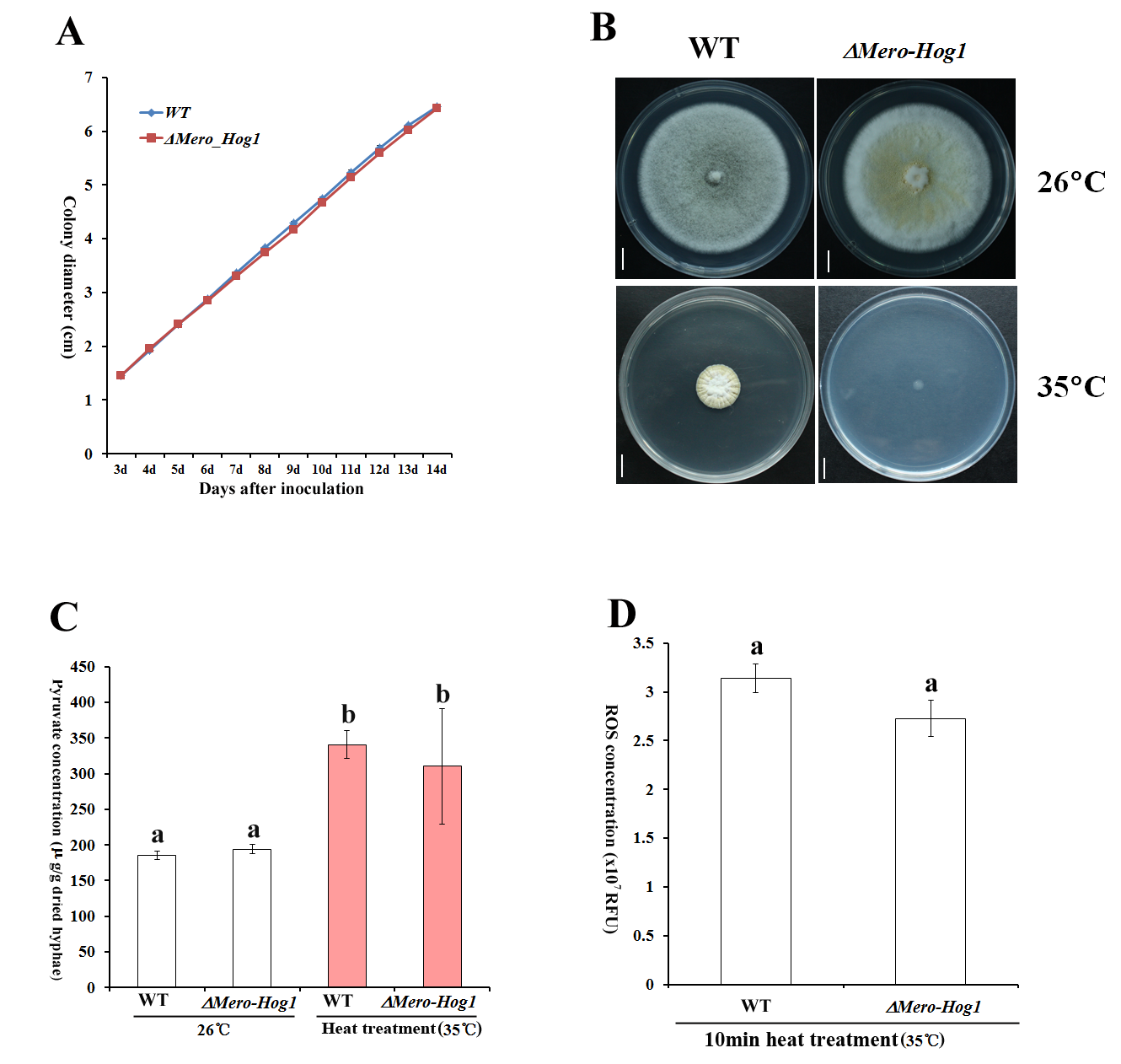

Supplement: FIG S8 [file mbo004173466sf8.tif]

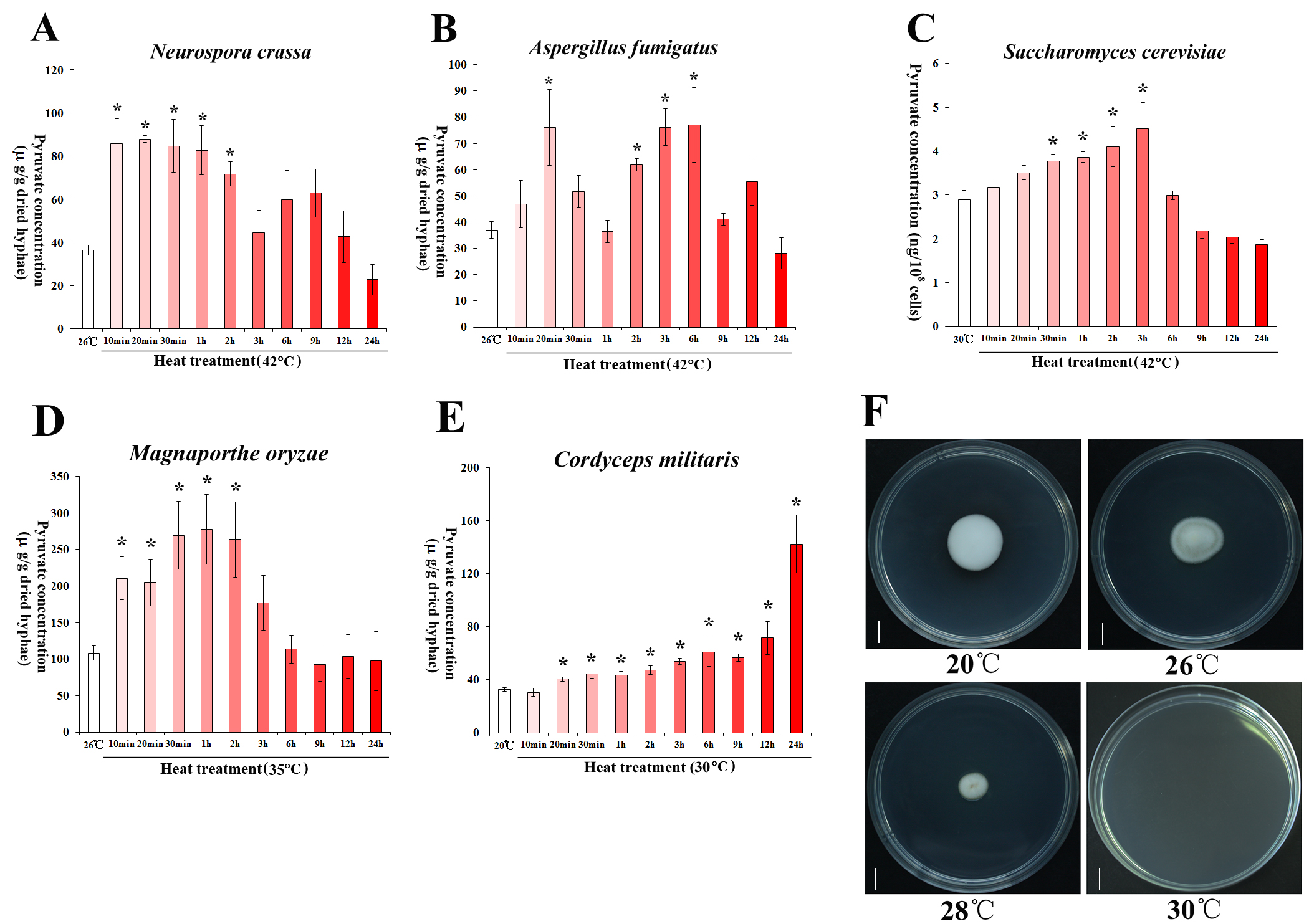

Supplement: FIG S9 [file mbo004173466sf9.jpg]
