# Supplementary material for: Pyruvate Accumulation Is the First Line of Cell Defense against Heat Stress in a Fungus
Source: mBio. 2017 Sep 5;8(5):e01284-17. doi: 10.1128/mBio.01284-17 (PMC5587913; doi:10.1128/mBio.01284-17)
Supplement: TABLE S1 [file mbo004173466st1.pdf]

Table S1 The list of genes analyzed by qRT-PCR in this study

Functions	Gene Name	Function	Forward primer	Reverse primer
Glycolysis	MAA_04209	Hexokinase	ATTCAAAGAACGTGGTGCCC	ATTGCCAGCCTTGACTCTCT
	MAA_05057	Hexokinase	GTCACTTCAAGCCGCTACAG	CAACTCGCGAATCTCTGACG
	MAA_09075	Phosphofructokinase	TTGCCAACATTATCCGCGAG	CTTCTTGACGCGGTTATGGG
	MAA_06851	Pyruvate kinase	TGTCTGGTGAAACGGCAAAG	CAATGATACCACCAGCAGCC
Pyruvate permease	MAA_02781	Transport pyruvate from cytoplasma into mitochondira	TCATCCCCATCCACCTCATG	TAGACGCAGCCCATGAAGAT
Pyruvate dehydrogenase complex	MAA_08787	Pyruvate dehydrogenase E1 component beta subunit	GTCAACTCTGCCGGAAAGAC	CAGCCTTGAGAAGACCCTTG
	MAA_03599	Mitochondrial substrate carrier	GTTAACGGCATGGACGTCTT	CCTCACGGGTACGGTAGGTA
	MAA_02750	Pyruvate dehydrogenase complex	GTGGAGAACGATGATGGCAC	AGAAGCAGCTCAAGAGGGTT
Tricaboxylic Acid Cycle (TCA cycle)	MAA_04490	Dihydrolipoyl dehydrogenase(a subunit of α-ketoglutarate dehydrogenase complex)	TCTCGTCAAAATGCTTGCAG	CTTCCGCTAATGTTGGGTGT
	MAA_01828	Citrate synthase	ACAAGAAGGAGTACTGGGGC	GGTTGGCGAAGTTGAAGGAG
	MAA_03118	Isocitrate dehydrogenase	TAAGAATCCGGTCGTGGAAC	GTGACCTGGTCGTTGGTCTT
	MAA_09446	Malate dehydrogenase	AAGGCCTAGTCACCAAGGAC	CAACCGAAGCCTTCAAAGCT
Produce oxaloacetic acid(OAA)to supplement to the TCA cycle	MAA_04366	Pyruvate carboxylase	TCGGGAGATATGCTGAACCC	ACACCAGTACCAGCAGAGTC
Acetaldehyde synthesis	MAA_09838	Thiamine pyrophosphate enzyme	CACGGGCTTGACATTGTGAT	TGTTCACCTCGTCATTCCCA
Fermentation (lactate synthesis from pyruvate)	MAA_09321	Lactate dehydrogenase	GTAATGTTCGTCCCGTCAGC	CCATCAGCAAGCACATGAGT
	MAA_07000	D-lactate dehydrogenase	TGCCTACGACCCATTTCCAT	TATTCACCAGCATCACCCCA
Glyoxylate cycle	MAA_01023	Isocitrate lyase	TACATTGACTACCTCCGGCC	CAGCCCATAACGTCCCATTG
	MAA_04402	Isocitrate lyase	CCGTCATGAAGCTCACCAAG	AGGTCCGTGCCCATAATGTC
Leu,Ile,Val synthesis	MAA_07650	Acetolactate synthase small subunit	AACTGCTCTCTCACCATCGT	ATGTCCAAAACCTTGCCACC
ROS scavengers	MAA_00836	Superoxide dismutase	GTCACCTTCGAGCAGGACTC	CGTGAGGGTTGAAGTGAGGT
	MAA_02494	Copper chaperone for superoxide dismutase	GAGGCTCTGCCTTTGTTGAC	TCCTCCCACAGTGTCTTTCC
	MAA_04749	Iron superoxide dismutase	CGGATCGTGACTACCAAGGA	GAACACATCATCGCGTCCTC
	MAA_10313	Cu/Zn superoxide dismutase	ATAGCAGGGCCAAGGATTCC	GTGATCCTCGTCTTGTTCGC
	MAA_04142	Cu/Zn superoxide dismutase	GCGATTTGAGAACTTGCCCA	ACAATGAAGGGGTCGCTAGT
	MAA_04372	Fe superoxide dismutase	AAGAACCAGGTCTACGGCAG	GGCAACCTTTTCCCAGTCAA
	MAA_05433	Manganese/iron superoxide dismutase	CTCTTGTCACTCGTGCCAAC	AGTGCTCCAGTTGATGACCT
	MAA_05879	Catalase	ATCAACAGACCCGTTGTTCC	GTGAAGAAGCCTTTGCCTTG
	MAA_10436	Catalase	ACTTTCAACTTCCGCCGAAC	TCCCCGTCCACATTTCATGA
	MAA_10327	Peroxisomal catalase	CGATATTGGGGCACTGAAGC	ACGGGTTCCAGATCTCCTTG
	MAA_09564	Catalase	CGATTTGCTCCCCTCAACAG	CGTACGTCTTGAAGCCCATG
	MAA_06740	Catalase	TCCACTGGTTCCTGTCATCC	GCATCTTCGGGGTCAATGAC
	MAA_05785	Catalase	GAGATGGTGGCATGGTTGTC	CGTCACATCGCCAAAGAGAG
	MAA_05543	Catalase	GTGACGGAGGAGGACTTTGA	TCGACCCGACTAAACAACGA

	MAA_03203	Catalase	GTTTGGCCACCTCAAGACTG	GCACAACCTCCTTCAAGCTC
	MAA_01261	Catalase	ATTGCCCATTCCTTCAACGG	CTGCCTGTTCAAAATCCGCT
	MAA_06893	Peroxiredoxins	TGACCCCAAGAAGACCATCC	CTTGATGATGCGGAGGTTGG
	MAA_01511	Glutathione peroxidase	GTACCCCGATGACTTCACCA	CCATTCGTACAAGTCGCTGG
TRX-TRR & GRX-GLR	MAA_09053	Thioredoxin	TGCCGATGGTCTCTTCAAGA	CTCTGCGGCGCTTTATGAAT
	MAA_04990	Thioredoxin-like fold protein	CAGGTTCGGTGGTAAAACGG	GGTTCTCAGCGTCTCGTAGT
	MAA_00709	Thioredoxin reductase	ACCGTCACAAAGCTAGACCT	AGATGCCGTTTTGCCAGTAC
	MAA_02039	Glutathione S-transferase	ATTGACCAGTTCCCGTCCAT	CCGTATTGCTTCTTGGCCTC
	MAA_04710	Glutathione S-transferase	CGTACTTTGGCCAGCTCATC	GGGACAAACATGAGATCGGC
Heat shock proteins	MAA_03832	HSP (Heat shock protein)	AGGTCCTGCAGCTCATTTCT	CCGGCAAACTCGTAGTCATG
	MAA_10381	HSP (Heat shock protein)	CGCCCAAATAAAGGAGCCAG	TAACGGCACCCTGATCAACT
	MAA_07190	HSP (Heat shock protein)	ACATTGAATTCACCGAGCCG	CAGTACTTGTACTTGGGCGC
